# Physiological specificity of muscle-strengthening activity: a comparative analysis of associations with arthritis and cardiovascular disease prevalence in U.S. adults (NHIS 2024)

**DOI:** 10.1016/j.pmedr.2026.103495

**Published:** 2026-05-08

**Authors:** Dennis Anheyer, Thomas Ostermann, Holger Cramer

**Affiliations:** a Institute of General Practice and Interprofessional Care, University Hospital Tuebingen, Tuebingen, Germany; b Robert Bosch Center for Integrative Medicine and Health, Bosch-Health-Campus, Stuttgart, Germany; c Department of Psychology and Psychotherapy, Faculty of Health, Witten/Herdecke University, Witten, Germany

**Keywords:** Muscle-strengthening activity, Resistance training, Arthritis, Epidemiology, NHIS

## Abstract

**Objective:**

Physical activity guidelines recommend both aerobic physical activity (APA) and muscle-strengthening activity (MSA) for chronic disease prevention. MSA benefits musculoskeletal health, whereas APA supports cardiovascular health, but epidemiological studies seldom separate MSA-specific associations from those of APA. We examined whether MSA relates more strongly to arthritis than to cardiovascular disease (CVD).

**Methods:**

We analyzed 31,238 non-institutionalized U.S. adults from the 2024 National Health Interview Survey in a cross-sectional design. Outcomes were self-reported physician diagnoses of arthritis and CVD. We fitted separate survey-weighted logistic regression models for arthritis and CVD, adjusting for APA, age, sex, race/ethnicity, body mass index, and smoking.

**Results:**

Each additional MSA day per week was associated with lower arthritis prevalence (adjusted odds ratio [OR] 0.92, 95% confidence interval [CI] 0.88–0.96). No association was observed for CVD (OR 0.98, 95% CI 0.92–1.04). The formal comparison indicated a significantly stronger association of MSA with arthritis than with CVD (*P* = 0.027).

**Conclusions:**

MSA was associated with lower arthritis prevalence but not with CVD, despite adjustment for APA and major confounders. These findings are compatible with more salient musculoskeletal than cardiovascular benefits of MSA. Promoting MSA alongside aerobic activity may support joint health and functional mobility across adulthood.

## Highlights

• Each extra strength-training day/week related to lower arthritis prevalence.
• Strength training showed no association with cardiovascular disease.
• A formal coefficient comparison supported stronger links for arthritis than heart disease.
• Survey-weighted logistic models adjusted for aerobic activity and key confounders.

## 1 Introduction

Physical inactivity remains a pervasive, leading modifiable risk factor underlying the escalating global prevalence of non-communicable diseases (NCDs) (Lee et al., 2012; Guthold et al., 2020). The World Health Organization (2023) estimates that over 1.4 billion adults fail to meet recommended levels of physical activity, contributing annually to approximately 5 million premature deaths worldwide (World Health Organization (WHO), 2022). To address this challenge, major health bodies, including the U.S. Department of Health and Human Services, strongly advocate for adherence to the Physical Activity Guidelines for Americans (Piercy et al., 2018).

Both aerobic physical activity (APA) and muscle-strengthening activity (MSA) are recommended: While APA leads to enhanced cardiorespiratory fitness and protection against diseases such as hypertension and coronary heart disease (Ashton et al., 2020), MSA preserves skeletal muscle mass and bone density, positively influencing age-related disorders like sarcopenia and arthritis (Haynes et al., 2020; Lo et al., 2024; Cheng et al., 2022). Recent meta-analyses underline that not only the frequency but also the regular integration of MSA into daily routines is crucial for sustainable preventive effects (Jochem et al., 2019; Cho et al., 2023).

However, despite the clear health benefits and the increasing body of evidence supporting MSA, the methodological approach used in most large-scale epidemiological studies still lacks a focus on physiological specificity; that is, a direct statistical comparison between the protective effects of APA and MSA on different chronic disease endpoints is rarely conducted (Momma et al., 2022; Stamatakis et al., 2018). The 2024 Global Burden of Disease report, for instance, highlights MSA's global prevalence but does not address organ-system-specific differences in impact (Xu et al., 2022).

This oversight restricts the translation of scientific knowledge into practice, as nuanced and disease-specific recommendations remain underdeveloped. Against this background, the present study seeks to fill this gap by directly comparing the independent associations of MSA with arthritis/joint problems versus CVD among U.S. adults, utilizing representative complex survey data. In doing so, the work aims to improve the precision of public health advice and contribute to the optimization of prevention strategies that are tailored to different risk profiles and disease mechanisms (Tsao et al., 2023; Xu and Wu, 2021).

## 2 Methods

### 2.1 Study design and data source

This study employs a cross-sectional design utilizing publicly available data from 2024 National Health Interview Survey (NHIS). The NHIS is an annual, nationally representative survey of the non-institutionalized civilian population of the United States, conducted by the National Center for Health Statistics (NCHS). The survey employs a complex, multi-stage probability sampling design, which necessitates the use of sampling weights (WTFA_A) and design variables (PPSU and PSTRAT) for all statistical estimations to account for non-response and complex variance estimation.

### 2.2 Study population and variable operationalization

The study focused on the presence of two distinct groups of chronic conditions, operationalized as binary variables (Yes/No) as dependent variables (Outcomes:

1. Arthritis/Joint Problems (Musculoskeletal Outcome): Defined as a positive self-report to the question: “Has a doctor or other health professional ever told you that you had Arthritis?” (NHIS Variable Name: ARTHEV_A)
2. Cardiovascular Disease (CVD) Prevalence: Defined as a positive self-report to the diagnosis of Coronary Heart Disease (NHIS Variable Name: CHDEV_A) or Stroke (NHIS Variable Name: STREV_A)

As primary independent variables physical activity was assessed based on self-reported leisure-time activities:

1. Muscle-Strengthening Activity (MSA): Measured as a continuous variable representing the number of days per week the participant reported engaging in muscle-strengthening exercises (NHIS Variable Name: STRFREQW_A).
2. Aerobic activity: Measured as a continuous variable representing the total weekly minutes of equivalent moderate-intensity aerobic physical activity (NHIS variables: MODMIN_a and VIGMIN_A). This variable was included as a crucial control

### 2.3 Covariates

All models were adjusted for Age (continuous), Sex, Race/Ethnicity, Body Mass Index (BMI) (Categorical), and Current Smoking Status.

### 2.4 Statistical analysis

Two separate, multivariable logistic regression models were conducted in R using the svyglm() function from the survey package to incorporate the design variables and final sampling weights.

Model A (Musculoskeletal Specificity): This model assessed the association between Muscle-Strengthening Activity (MSA) and the odds of Arthritis/Joint Problems. The model included Aerobic Activity as a control variable, along with all demographic and lifestyle covariates.

Model B (Cardiovascular Comparison): This model assessed the association between MSA and the odds of CVD Prevalence. This model included the same set of independent and control variables as Model A to ensure direct comparability.

The core hypothesis, testing whether the protective association of MSA is specific to the musculoskeletal outcome, was tested by directly comparing the estimated Odds Ratios (ORs) for MSA obtained from Model A and Model B. The difference between the two log-odds coefficients was formally tested using a Wald *Z*-test derived from the survey package output. A statistically significant difference (*P* < 0.05) would indicate that the effect specificity is statistically supported.

### 2.5 Sensitivity Analysis

To ensure the robustness of the primary findings, two key sensitivity analyses were conducted: 1) Categorical Exposure: MSA was re-categorized into a binary variable (meeting guidelines: ≥2 days/week vs. not meeting guidelines: <2 days/week), and 2) Outcome Refinement: CVD was narrowly defined to include only Coronary Heart Disease (excluding Hypertension) to test for potential confounding.

### 2.6 Power considerations

Given the large sample size of the NHIS 2024 (31,238 fully complete cases), the study is expected to have sufficient statistical power to detect small to moderate effects for the outcomes examined.

### 2.7 Disclosure of ethical compliance

This study was based on a secondary analysis of publicly available, de-identified data from the 2024 National Health Interview Survey (NHIS). In accordance with institutional requirements for research using anonymized public-use datasets, no separate ethical approval or formal exemption was required, as no directly identifiable human participant data were accessed or analyzed.

### 2.8 Statistical software

All statistical analyses were performed exclusively using the R statistical computing software (4.4.1). R was selected because its dedicated packages, notably the survey package (Lumley, 2004), allow for the accurate accounting of the NHIS's complex sampling design (including stratification, clustering, and weighting). The use of these advanced methods is crucial to obtain unbiased point estimates and correct standard errors, which ensure the representativeness of the results for the target population.

## 3 Results

### 3.1 Sample characteristics and disease prevalence

The overall weighted prevalence of diagnosed Arthritis in the study cohort was 21.29% (SE 0.30), while the prevalence of a previous Cardiovascular Disease (CVD) diagnosis was 7.18% (SE 0.17).

The mean age of the overall cohort was 48.40 years (SD 18.71), and 51.32% were female. The majority of participants were non-Hispanic White (62.49%). Nearly one-third of the population fell into the Morbid Obese category (33.37%).

### 3.2 Comparison by arthritis status

Demographic and health characteristics stratified by Arthritis status are presented in Table 1. Significant differences (*P* < 0.01) were observed for nearly all variables, with the exception of physical activity measures.Age and Sex: Participants with Arthritis were significantly older (mean age 63.53 years, SD 14.59) than those without Arthritis (mean age 44.28 years, SD 17.56). The Arthritis group also had a higher proportion of females (57.74%) compared to the non-Arthritis group (49.58%).

**Table 1:** Demographic characteristics of adults in the 2024 National Health Interview Survey, United States, stratified by arthritis and cardiovascular disease status (CVD).

Variable	Category	Total (Mean/%) (SD)	ArthritisYes (SD)	ArthritisNo (SD)	*P*-Value (Arthritis)	CVD Yes (SD)	CVD No (SD)	P-Value (CVD)
**Age, mean (SD)**	–	48.40 (18.71)	63.53 (14.59)	44.28 (17.56)	<0.01	67.74 (13.76)	46.85 (18.19)	<0.01
**Sex,** **% (SD)**	Female	51.32 (49.98)	57.74 (49.40)	49.58 (50.00)	<0.01	41.47 (49.27)	52.10 (49.96)	<0.01
	Male	48.68 (49.98)	42.26 (49.40)	50.42 (50.00)	<0.01	58.53 (49.27)	47.90 (49.96)	<0.01
**Race/** **Ethnicity, % (SD)**	Hispanic	18.33 (38.69)	9.84 (29.79)	20.66 (40.49)	<0.01	10.47 (30.62)	18.97 (39.21)	<0.01
	Non-Hispanic AIAN	0.66 (8.09)	0.77 (8.74)	0.63 (7.90)	<0.01	0.73 (8.52)	0.65 (8.05)	<0.01
	Non-Hispanic Asian	6.43 (24.52)	3.70 (18.88)	7.16 (25.78)	<0.01	4.29 (20.26)	6.59 (24.81)	<0.01
	Non-Hispanic Black	12.10 (32.61)	11.84 (32.31)	12.17 (32.70)	<0.01	12.71 (33.31)	12.06 (32.57)	<0.01
	Non-Hispanic White	62.49 (48.42)	73.85 (43.95)	59.38 (49.11)	<0.01	71.80 (45.00)	61.72 (48.61)	<0.01
**BMI Category, % (SD)**	Morbid Obese	33.37 (47.15)	42.41 (49.42)	30.92 (46.22)	<0.01	37.56 (48.43)	33.03 (47.03)	<0.01
	Obese	33.57 (47.22)	32.27 (46.75)	33.94 (47.35)	<0.01	34.32 (47.48)	33.52 (47.21)	<0.01
	Overweight	31.29 (46.37)	23.89 (42.64)	33.28 (47.12)	<0.01	26.26 (44.00)	31.69 (46.53)	<0.01
	Under/ Normal	1.77 (13.18)	1.43 (11.89)	1.86 (13.51)	<0.01	1.87 (13.54)	1.76 (13.14)	<0.01
**Smoking Status, % (SD)**	Current	9.94 (29.91)	13.02 (33.65)	9.11 (28.78)	<0.01	12.55 (33.13)	9.73 (29.64)	<0.01
	Former	22.42 (41.71)	34.64 (47.58)	19.11 (39.32)	<0.01	39.47 (48.88)	21.08 (40.79)	<0.01
	Never	67.64 (46.78)	52.34 (49.95)	71.78 (45.01)	<0.01	47.98 (49.96)	69.19 (46.17)	<0.01
**MSA Days/Weekmean (SD)**	–	3.30 (1.88)	3.26 (1.90)	3.30 (1.88)	0.41	3.35 (2.00)	3.29 (1.87)	0.58
**Aerobic Min/Weekmean (SD)**	–	122.36 (92.77)	122.82 (99.98)	122.27 (91.72)	0.86	134.47 (115.20)	121.95 (91.94)	0.06

Abbreviations.CVD - . Cardiovascular Disease; MSA - Muscle Strengthening Activity, SD – Standard Deviation.

Race/Ethnicity: Non-Hispanic White individuals were substantially overrepresented in the Arthritis group (73.85%) compared to the non-Arthritis group (59.38%). Conversely, the proportion of Hispanic individuals was higher in the non-Arthritis group (20.66%) than in the Arthritis group (9.84%).

BMI and Smoking: The prevalence of Morbid Obesity was markedly higher among individuals with Arthritis (42.41%) versus those without (30.92%). Similarly, the Arthritis group reported a higher rate of being Former smokers (34.64% vs. 19.11%).

Physical Activity: There were no statistically significant differences in the average MSA Days per Week (3.26 vs. 3.30, *P* = 0.410) or Aerobic Minutes per Week (122.82 vs. 122.27, *P* = 0.860) between the two groups.

### 3.3 Comparison by cardiovascular disease (CVD) status

Significant differences were also observed across all demographic and health characteristics when comparing individuals with and without CVD (*P* < 0.001 for all categorical variables).

Age and Sex: The CVD group was significantly older (mean age 67.74 years, SD 13.76) compared to the non-CVD group (mean age 46.85 years, SD 18.19). Unlike the Arthritis comparison, the CVD group showed a higher proportion of males (58.53%) and a lower proportion of females (41.47%) than the non-CVD group.

Race/Ethnicity and BMI: The CVD group had a higher prevalence of non-Hispanic White individuals (71.80%) and individuals classified as Morbid Obese (37.56%) compared to the non-CVD group (61.72% and 33.03%, respectively).

Smoking Status: The largest difference in smoking was observed in the Former smoker category (39.47% in the CVD group vs. 21.08% in the non-CVD group).

Physical Activity: No significant difference was found for MSA Days per Week (*P* = 0.578). However, Aerobic Minutes per Week showed a near-significant difference (134.47 vs. 121.95, *P* = 0.059), suggesting individuals with CVD reported slightly more aerobic activity.

### 3.4 Association between muscle strengthening activity and health outcomes

In survey-weighted logistic regression models adjusted for aerobic activity, age, sex, BMI, and smoking, greater frequency of muscle-strengthening activity (MSA) was associated with a significantly lower likelihood of arthritis (Model A: OR = 0.92 per additional day per week; 95% CI 0.88, 0.96). In contrast, MSA frequency was not significantly associated with cardiovascular disease prevalence (Model B: OR = 0.98; 95% CI 0.92, 1.04). The Wald test comparing the MSA coefficients between the two models indicated a statistically significant difference (Z = 2.21; *p* = 0.03), supporting disease-specificity of the protective effect for musculoskeletal rather than cardiovascular outcomes.

In the categorical sensitivity analysis using the recommended guideline cutoff (≥ 2 days/week), MSA participation remained protective for arthritis (OR = 0.81; 95% CI 0.73, 0.89) but showed no association with CVD (OR = 0.96; 95% CI 0.84, 1.10). A second sensitivity model, restricting CVD to coronary heart disease only, yielded similar null findings (OR = 0.97; 95% CI 0.90, 1.05). The adjusted associations between MSA and the two respective health outcomes are detailed in Table 2.

**Table 2:** Adjusted odds ratios for the association between muscle-strengthening activity (MSA) and arthritis/cardiovascular disease status among adults in the 2024 National Health Interview Survey.

Model	Outcome	MSA Effect (per +1 day/week)	Adjusted Odds Ratio (OR) (95% CI)	p-value
**A**	Arthritis (ARTHEV_A)	−0.09 (SE = 0.02)	**0.92 (0.88, 0.96)**	< 0.01
**B**	Cardiovascular Disease (CVD) (CHDEV_A or STREV_A)	−0.02 (SE = 0.03)	**0.98 (0.92, 1.04)**	0.54
	**Wald Test (Model A vs B)**	Z = 2.21	p = 0.03	–
**Sensitivity 1**	MSA ≥ 2 days/week vs < 2 days	Arthritis OR = 0.81 (0.73, 0.89) • CVD OR = 0.96 (0.84, 1.10)	–	–
**Sensitivity 2**	CVD = Coronary Heart Disease only	–	**0.97 (0.90, 1.05)**	0.46

Note. Survey-weighted logistic regression models adjusted for age, sex, aerobic activity (equivalent moderate minutes per week), BMI category, and current smoking. Estimates incorporate strata (PSTRAT), PSU (PPSU), and final weights (WTFA_A). Abbreviations.CI – Confidence Interval, CVD - . Cardiovascular Disease; MSA - Muscle Strengthening Activity, OR – Odds Ratio, SE – Standard Error.

Collectively, these results indicate that muscle-strengthening activity is specifically associated with reduced odds of arthritis, independent of aerobic activity and demographic factors, whereas no corresponding association was observed for cardiovascular disease prevalence.

## 4 Discussion

This study used nationally representative data from the 2024 National Health Interview Survey (NHIS) to investigate the association between Muscle Strengthening Activity (MSA) and two major chronic conditions—arthritis and cardiovascular disease (CVD)—while rigorously accounting for the complex sampling design. All models adjusted for age, sex, BMI, smoking status, race/ethnicity, and total aerobic physical activity.

The principal finding of this analysis is the significant and independent protective association between MSA and prevalent arthritis, rather than CVD. Each additional day of MSA per week was associated with an 8% lower odds of having been diagnosed with arthritis (Adjusted OR = 0.92, *p* < 0.01). In contrast, the association between MSA and prevalent CVD was statistically non-significant (Adjusted OR = 0.98, *p* = 0.54). A formal Wald test comparing the magnitude of these effects revealed a statistically significant difference (p = 0.03), suggesting that the protective effect of MSA is specific to musculoskeletal outcomes rather than cardiovascular ones.

The beneficial role of muscle-strengthening activities (MSA) for musculoskeletal health is well established (Lim et al., 2024; Vincent and Vincent, 2012; Zeng et al., 2021). Randomized trials and meta-analyses in people with osteoarthritis demonstrate that regular resistance training reduces pain and improves joint function, muscle strength, and physical performance, thereby helping to limit functional decline associated with arthritis (Lim et al., 2024; Vincent and Vincent, 2012). Proposed mechanisms include enhanced periarticular muscle strength and joint stability, reduction of local inflammation, and beneficial effects on cartilage and subchondral bone metabolism (Zeng et al., 2021). Nevertheless, population-based evidence on the primary prevention of arthritis through MSA remains relatively limited. In this context, the present findings add epidemiological support for MSA as a potentially protective factor against arthritis, consistent with observational data linking a history of strength training to lower odds of knee pain and radiographic knee osteoarthritis, even after adjustment for other lifestyle factors (Lo et al., 2024).

In contrast, epidemiological research on cardiovascular disease (CVD) prevention has yielded more heterogeneous results for MSA. Resistance training interventions consistently improve cardiometabolic risk markers, including reductions in resting blood pressure, improvements in insulin sensitivity, and favourable changes in lipid and inflammatory profiles (Cornelissen et al., 2011; Costa et al., 2019; Li et al., 2021). However, a recent meta-analysis of cohort studies suggests that the association between MSA and clinical CVD events or mortality is modest, often showing a J-shaped dose–response and a strong dependence on training volume and concurrent aerobic activity, with some cohorts finding no additional benefit of MSA alone beyond aerobic exercise (Momma et al., 2022). In line with this pattern, our study did not detect a statistically significant cross-sectional association between MSA frequency and prevalent CVD. This absence of association may reflect the long latency of atherosclerotic disease and the possibility that substantial, sustained exposure to resistance training is required to translate intermediate cardiometabolic improvements into detectable differences in CVD prevalence (Momma et al., 2022; Cornelissen et al., 2011; Costa et al., 2019; Li et al., 2021).

More broadly, the present findings should be interpreted in the context of evidence showing that muscular strength is associated with cardiovascular outcomes, particularly heart failure, and that even low-frequency resistance exercise may be linked to lower all-cause and CVD mortality independent of aerobic activity (Carbone et al., 2020; Laukkanen et al., 2021; Liu et al., 2019). In parallel, higher aerobic fitness has been associated with lower mortality in individuals with musculoskeletal conditions, and running has been linked to lower risks of hip and knee arthritis as well as lower cardiovascular and all-cause mortality (Williams, 2013; Lavie et al., 2015). Taken together, this broader literature suggests that different domains of physical activity, including muscle-strengthening and aerobic exercise, may contribute to musculoskeletal and cardiovascular health through partly distinct but complementary pathways.

The finding of a selective association between MSA and arthritis is compatible with the idea that musculoskeletal adaptations to resistance training may be more readily detectable than systemic cardiovascular changes in cross-sectional analyses (Turner et al., 2019; Wang et al., 2026; Green et al., 2017). Randomized trials and meta-analyses indicate that resistance training can improve pain, muscle strength and physical function in knee osteoarthritis within weeks to months, suggesting relatively rapid neuromuscular and structural changes at the joint level (Turner et al., 2019; Wang et al., 2026). By contrast, experimental and translational work on vascular adaptation shows that functional and structural changes in the arterial wall, and their impact on atherosclerotic disease, tend to develop over longer time scales (Green et al., 2017). Such differences in adaptation dynamics may contribute to a clearer cross-sectional signal for MSA in relation to arthritis than for cardiovascular disease.

Furthermore, individuals with established cardiovascular disease are often advised to modify their activity patterns and may self-limit certain forms of exercise, so that lower MSA levels among those with CVD could attenuate or obscure any protective association in cross-sectional analyses (reverse causality) (Sattar and Preiss, 2017).

The observed disease-specific pattern therefore tentatively supports the view that MSA may have particularly salient effects on joint and musculoskeletal outcomes, complementing its established therapeutic role in symptomatic arthritis (Wang et al., 2026; Kolasinski et al., 2020; Osthoff et al., 2018). This interpretation is consistent with contemporary recommendations from the American College of Rheumatology/Arthritis Foundation and EULAR, which identify structured exercise, including muscle-strengthening activities, as a core non-pharmacological component of osteoarthritis and inflammatory arthritis management (Kolasinski et al., 2020; Osthoff et al., 2018).

The key strengths of this study include its national representativeness, large sample size, and use of complex survey-weighted logistic regression, which ensures accurate variance estimation. Adjusting for aerobic activity allowed isolation of the specific role of MSA.

However, as a cross-sectional analysis, causality cannot be inferred. Individuals with existing CVD or arthritis may alter their physical activity due to medical advice or symptom limitation, introducing reverse causation. Additionally, both MSA and disease diagnoses were self-reported, which may lead to measurement error or recall bias. Despite these limitations, the internal consistency of the arthritis findings across sensitivity analyses supports their robustness.

From a clinical and public-health perspective, these findings emphasize MSA as an essential component of musculoskeletal disease prevention and management. Regular muscle-strengthening activity (≥ 2 days per week) should be promoted broadly, not only for its well-documented functional benefits in individuals with arthritis but also as a potential primary preventive measure against its onset. For cardiovascular prevention, MSA should continue to be encouraged as part of a combined exercise regimen, though aerobic activity remains the cornerstone for reducing CVD risk.

Future studies should utilize longitudinal designs to confirm the temporality of associations and explore whether consistent, long-term participation in MSA contributes to both musculoskeletal and cardiometabolic health outcomes. Objective measurement of physical activity (e.g., via accelerometry) would help minimize reporting bias. Moreover, biomarker studies assessing inflammatory and metabolic mediators (e.g., myokines such as IL-6 and irisin) may clarify the mechanistic pathways linking MSA to differential health effects.

## 5 Conclusion

This nationally representative analysis suggests that muscle-strengthening activity is associated with lower odds of arthritis, whereas no clear association was observed with cardiovascular disease in U.S. adults. Taken together, these findings are consistent with a more pronounced musculoskeletal relevance of MSA, while remaining in line with its broader functional and therapeutic value. Encouraging MSA alongside aerobic exercise therefore appears to be a reasonable component of strategies aimed at comprehensive chronic disease prevention and healthy aging.

## Availability of data and materials

The data supporting the findings of this study are publicly available from the U.S. National Center for Health Statistics (NCHS) National Health Interview Survey (NHIS) 2024 public-use release. We analyzed the NHIS 2024 Sample Adult public-use data file (with the accompanying codebook and survey design documentation) available from the CDC/NCHS NHIS 2024 documentation page.

The analytic dataset used in this article represents the minimal dataset necessary to reproduce the results and was created by subsetting eligible adult respondents and recoding variables from the NHIS 2024 public-use file(s) as described in the Methods; no new primary data were generated.

## Author contribution

DA conceived and designed the study, conducted the statistical analyses, drafted the manuscript, and interpreted the data. TO and HC contributed to the analysis and interpretation of the data and critically revised and edited the manuscript for important intellectual content. All authors approved the final version of the manuscript and agree to be accountable for all aspects of the work.

## CRediT authorship contribution statement

**Dennis Anheyer:** Writing – review & editing, Writing – original draft, Visualization, Methodology, Formal analysis, Conceptualization. **Thomas Ostermann:** Writing – review & editing, Methodology, Conceptualization. **Holger Cramer:** Writing – review & editing, Supervision, Methodology, Conceptualization.

## Consent for publication

Not applicable.

## Ethics approval and consent to participate

Not applicable.

## Funding

This research received no specific grant from any funding agency in the public, commercial, or not-for-profit sectors.

## Declaration of competing interest

The authors declare the following financial interests/personal relationships which may be considered as potential competing interests: Holger Cramer and Thomas Ostermann reports a relationship with Journal of Integrative and Complementary Medicine that includes: board membership. If there are other authors, they declare that they have no known competing financial interests or personal relationships that could have appeared to influence the work reported in this paper.

## Data Availability

Data is freely availible.
